# Timing of follow-up blood cultures for community-onset bacteremia

**DOI:** 10.1038/s41598-019-51032-z

**Published:** 2019-10-10

**Authors:** Ching-Chi Lee, Chao-Yung Yang, Chih-Chia Hsieh, Ming-Yuan Hong, Chung-Hsun Lee, Hung-Jen Tang, Wen-Chien Ko

**Affiliations:** 1 Department of Adult Critical Care Medicine, Tainan Sin-Lau Hospital, Tainan, Taiwan; 2 Graduate Institute of Medical Sciences, College of Health Sciences, Chang Jung Christian University, Tainan, Taiwan; 3 Department of Emergency Medicine, National Cheng Kung University Hospital, Tainan, Taiwan; 4 Department of Medicine, National Cheng Kung University Medical College, Tainan, Taiwan; 5 Department of Medicine, Chi-Mei Medical Center, Tainan, Taiwan; 6 Department of Health and Nutrition, Chia Nan University of Pharmacy and Science, Tainan, Taiwan; 7 Department of Internal Medicine, National Cheng Kung University Hospital, Tainan, Taiwan

**Keywords:** Bacterial infection, Outcomes research

## Abstract

Bacteremia is associated with high morbidity and mortality, but the utility and optimal timing of follow-up blood cultures (FUBCs) remain undefined. To assess the optimal timing of FUBCs related to appropriate antibiotic therapy (AAT), adults with community-onset bacteremia and FUBCs after bacteremia onset were retrospectively studied during the 6-year period in two hospitals. Based on the time gap between the initiation of AAT and FUBC sampling, 1,247 eligible patients were categorized as FUBCs prior to AAT (65 patients, 5.2%), 0–3 days (202, 16.2%), 3.1–6 days (470, 37.7%), 6.1–9 days (299, 24.0%), and ≥9 days (211, 16.9%) after AAT. The prognostic impact of the growth of the same bacteria in FUBCs on 30-day mortality was evidenced only in patients with FUBCs at 3.1–6 days after AAT (adjusted odds ratio [AOR], 3.75; *P* < 0.001), not in those with FUBCs prior to AAT (AOR, 2.86; *P* = 0.25), 0–3 days (AOR, 0.39; *P* = 0.08), 6.1–9 days (AOR, 2.19; *P* = 0.32), and ≥9 days (AOR, 0.41; *P* = 0.41) of AAT, after adjusting independent factors of 30-day mortality recognized by the multivariable regression in each category. Conclusively, persistent bacteremia in FUBCs added prognostic significance in the management of adults with community-onset bacteremia after 3.1–6 days of AAT.

## Introduction

Bloodstream infections are associated with high morbidity and mortality despite the availability of potential antimicrobial therapy and advances in supportive care^1^. Blood culture is an essential tool for the isolation and characterization of causative pathogens in such infections. However, the principle of follow-up blood culture (FUBC) sampling was not “one-size-fits-all” in the literature. For example, it has been considered as a standard of care for patients with *Staphylococcus aureus* bacteremia^2,3^, but Gram-negative bacillary bacteremia was usually regarded to be transient or intermittent, and FUBCs were not routinely recommended^4,5^.

Furthermore, bacteremia may persist in FUBCs among patients with unresolved infections or receiving ineffective antimicrobial therapy^6,7^. Therefore, the result of FUBCs during antimicrobial therapy might indicate the prognostic impact. However, the question of “When should FUBCs be sampled after the administration of appropriate antibiotic therapy (AAT)?” has been debated. To achieve the optimal timing of FUBC sampling, we conducted a retrospective, multicenter study including a large cohort of adults with community-onset bacteremia. The aim was to determine the impact of the growth of FUBCs on patient outcomes at various time gaps between the FUBC sampling and AAT administration.

## Materials and Methods

### Study design and sites

This multicenter study was retrospectively conducted from January 2010 to December 2015 at two hospitals in southern Taiwan. One study hospital is a university-affiliated medical center with 1200 beds and another is a teaching hospital with 800 beds. Adult patients (aged ≥ 18 years) with community-onset bacteremia were included. The study was approved by the institutional review board of two hospitals and collectively reported by the format recommended by STROBE (Strengthening the Reporting of Observational Studies in Epidemiology)^8^.

### Patient population

During the study period, patients with blood cultures sampled at the emergency departments (EDs) were screened for bacterial growth in a computer database. Of adults with clinically significant bacteremia, clinical information was retrieved from medical charts. For community-onset bacteremia, patients were excluded if their blood culture results were regarded as being contaminated, showed fungal growth, or they were transferred from other hospitals. Furthermore, patients without FUBCs or those with nosocomial bloodstream infections, with un-standard procedures of blood-culture sampling, or with the uncertain fatality date, were excluded. Among the patients with multiple bacteremic episodes, only the first episode was taken into account. If a patient had several FUBCs during the same hospitalization, only the first one was considered. Based on the timings of blood cultures related to the initiation of AAT, eligible patients were categorized into five groups: FUBCs prior to AAT, 0–3 days, 3.1–6 days, 6.1–9 days, and >9 days of AAT.

### Data collection

By retrospective review of medical records, information collected in a predetermined case form included patient demographics and clinical characteristics, in terms of gender, age, bacteremia severity at onset, the type and severity of comorbidities, causative pathogens, bacteremia sources, image studies, prescribed antimicrobial agents, the duration of hospital stay and antimicrobial therapy, the date of defervescence, and surgical or radiological intervention. In addition, clinical outcomes at 30 days after bacteremia onset (*i.e*., ED arrival) regarded as the primary outcome of our study were recorded. Medical records were reviewed by two authors, and data discrepancy would be discussed for a consensus.

### Definitions

As previously defined, community-onset bacteremia indicated that the place of bacteremia onset was the community, and included healthcare-facility-acquired and community-acquired bacteremia^9,10^. Polymicrobial bacteremia was defined as the isolation of more than one microbial species in a bacteremic episode. Blood cultures with growth of potential contamination microorganisms, such as coagulase-negative staphylococci, *Micrococcus* species, *Bacillus* species, *Propionebacterium acnes*, or *Peptostreptococcus* species, were considered to be contaminated, based on the previous criteria^11^. With the exclusion of blood culture contamination, the FUBC growth of bacterial species different from initial pathogens was referred as nosocomial bacteremia. The period between bacteremia onset and the first FUBC sampling was regarded as the time-to-FUBC.

As previously described^9,10^, antimicrobial therapy was considered as appropriate if antibiotic route and dosage were administered as recommended in the Sanford Guide^12^ and causative pathogens were *in vitro* susceptible to the prescribed antibiotic(s) based on the breakpoints of the Clinical and Laboratory Standards Institute (CLSI) issued in 2016^13^. The time-to-AAT measured in hours was defined as the period between bacteremia onset and the initiation of AAT. A time-to-AAT of >24 hours was considered as inappropriate empirical therapy^10,14^.

Like previous definitions^15^, the removal of infected hardware, drainage of infected fluid collections, or resolution of obstruction for biliary or urinary sources was referred as adequate control of bacteremia source. Defervescence was defined as an afebrile state in which tympanic body temperature maintained at less than 37.0 °C for at least 24 hours^9^, and the time-to-defervescence as the period between the initiation of AAT and defervescence. The bacteremia severity was graded by the Pitt bacteremia score, a previously validated scoring system^10^. Comorbidities were defined as previous criteria^16^ and the comorbid severity was assessed by a delineated McCabe–Jackson classification, in which comorbidities were graded as being rapidly fatal, ultimately fatal, or non-fatal^17^.

### Microbiological methods

During the study period, standard procedures of blood-culture sampling included: (i) nurses performed blood sampling to collect two sets of blood cultures; (ii) each set of blood samples routinely consisted of one bottle for aerobic culture and another for anaerobic culture, with approximately 5–8 mL of blood in each bottle; and (iii) following sampling, culture bottles were immediately transported to the clinical laboratory department and incubated in a BACTEC 9240 instrument (Becton Dickinson Diagnostic Systems, Sparks, MD, USA) for five days at 35 °C. Bacteremic aerobic isolates were identified by the Vitek 2 system (bioMérieux, Durham, NC) and their antimicrobial susceptibility was determined by the disk diffusion method, based on the performance standards of CLSI in 2016^13^. Bacteremic isolates during the study period were prospectively stored for susceptibility testing, if the *in vitro* susceptibility of prescribed antimicrobial agents was not measured by the routine panel in the study hospital.

### Statistical analyses

Statistical analyses were performed by the Statistical Package for the Social Science for Windows (Version 20.0; Chicago, IL, USA). For the comparisons of category variables among different patient groups in varied time, Pearson chi-square test or Fisher exact, if the expected counts were less than 5, was used. Continuous variables were expressed as the mean values ± standard deviations and compared by the one-way analysis of variance among varied patient groups.

The impact of the growth of the same bacteria in FUBCs on 30-day mortality was studied by adjustment of independent predictors of 30-day mortality recognized in a hierarchical logistic regression model as the multivariable analysis, in which all the variables with a *P* value of less than 0.1 (or 0.2 for a small patient population) in the univariate analysis were included. A two-sided *P* value of less than 0.05 was considered to be significant.

### Ethic Approval

The study was approved by the institutional review board of National Cheng Kung University Hospital (ER-100-182, 5^th^ ed. revision) and Sin-Lau Hospital (SLH 9919-108-006), and the requirement of obtaining informed consent was all waived by two hospitals.

## Results

### Demographics and clinical characteristics of study cohort

**Figure 1:**
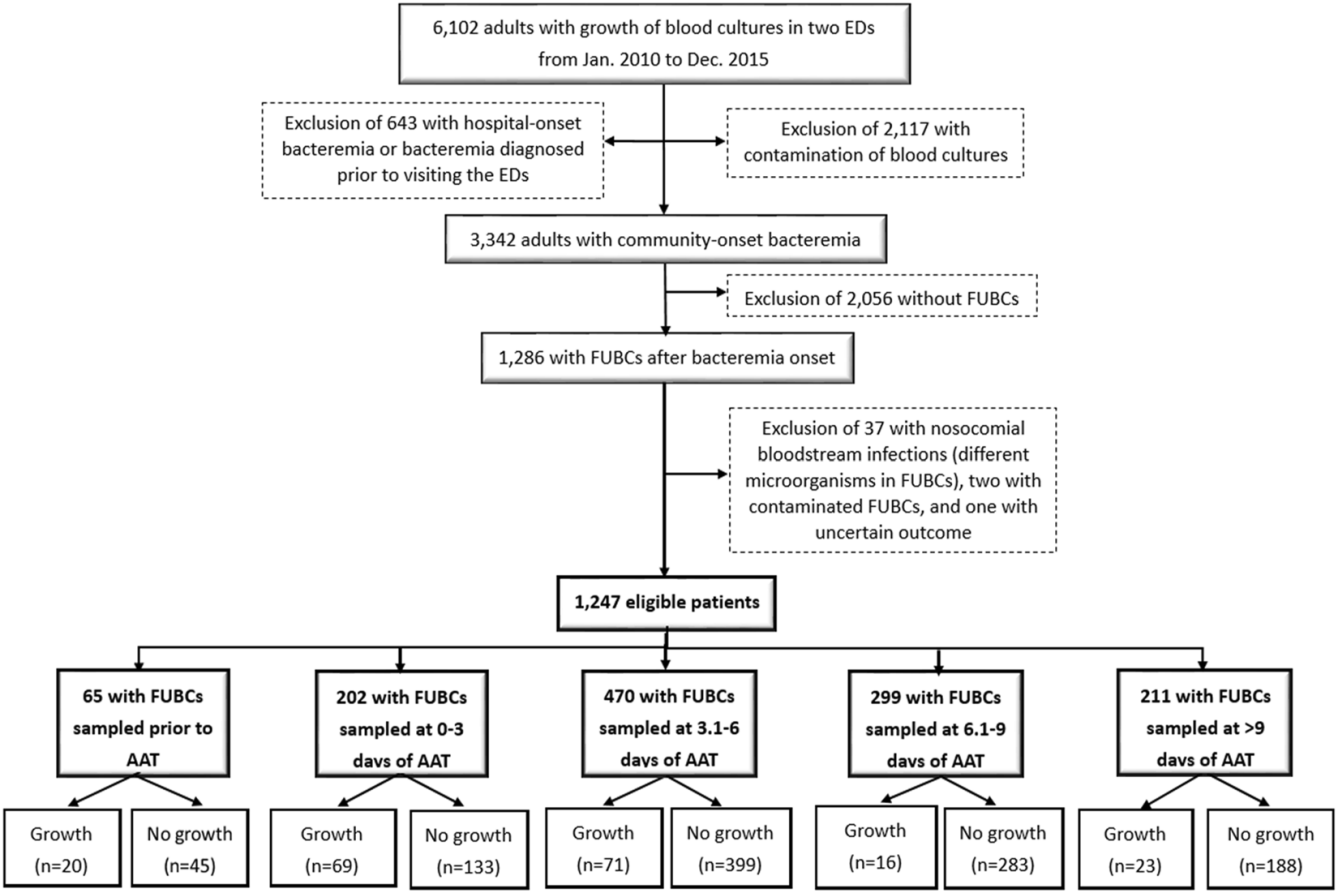
The flowchart of patient selection. AAT = appropriate antibiotic therapy; ED = emergency department; FUBC = follow-up blood culture.

A total of 1,247 adults were eligible based on the inclusion and exclusion criteria (Fig. 1). Their mean age was 66.9 years, and 698 (56.0%) were male. They had in average 1.8 FUBCs per patient (range, 1–9 blood cultures) and the median period between bacteremia onset and the first FUBC sampling was 6 days (interquartile range [IQR], 4–8 days). Their median (IQR) ED and hospital stay was 16.2 (6.0–28.1) hours and 19 (12–33) days. Patients with a critical illness (Pitt bacteremia score ≥ 4) at bacteremia onset accounted for 21.8% (272 patients). Overall crude 15-day and 30-day mortality rate was 6.3% (78 patients) and 12.6% (157), respectively.

Ten common sources of community-onset bacteremia in our study were urinary tract infections, pneumonia, skin and soft-tissue infections, intra-abdominal infections, bone and joint infections, primary bacteremia, infective endocarditis, vascular catheter-related infections, biliary tract infections, and liver abscess (Table 1). Because there were 128 episodes of polymicrobial bacteremia, a total of 1,409 isolates were identified and common aerobic pathogens included *Escherichia coli*, *Staphylococcus aureus*, *Streptococcus* species, *Klebsiella* species, *Enterococcus* species, *Pseudomonas* species, *Enterobacter* species, *Salmonella* species, *Acinetobacter* species, and *Proteus* species (Table 1).

**Table 1:** The proportion of community-onset bacteremia and bacterial growth in follow-up blood cultures (FUBCs), categorizied by bacteremia sources and causative pathogens.

Variables	Episode No. (% of total episodes)	No. of bacterial growth in FUBCs (%)
Bacteremia sources		
Urinary tract infections	260 (20.9)	29 (11.2)
Pneumonia	240 (19.2)	21 (8.8)
Skin and soft-tissue infections	186 (14.9)	23 (12.4)
Intra-abdominal infections	118 (9.5)	13 (11.0)
Bone and joint infections	103 (8.3)	35 (34.0)
Primary bacteremia	102 (8.2)	11 (10.8)
Infective endocarditis	90 (7.2)	36 (40.0)
Vascular-catheter related infections	77 (6.2)	30 (39.0)
Biliary tract infections	64 (5.1)	11 (17.2)
Liver abscess	42 (3.4)	3 (7.1)
Causative microorganisms		
*Escherichia coli*	324 (23.0)	37 (11.4)
*Staphylococcus aureus*	302 (21.4)	91 (30.1)
*Streptococcus* species	237 (16.8)	16 (6.8)
*Klebsiella* species	199 (14.1)	20 (10.1)
*Enterococcus* species	57 (4.0)	5 (8.8)
*Pseudomonas* species	51 (3.6)	6 (11.8)
*Enterobacter* species	33 (2.3)	12 (36.4)
*Salmonella* species	30 (2.1)	3 (10.0)
*Acinetobacter* species	22 (1.6)	6 (27.3)
*Proteus* species	20 (1.4)	2 (10.0)

### Growth of FUBCs in varied bacteremia sources and bacterial pathogens

Bacterial growth in FUBCs was most commonly found in the case of infective endocarditis with indicated FUBCs, and in terms of causative aerobic pathogens, bacterial growth in FUBCs was most often noted in the case of *Enterobacter* bacteremia (Table 1).

### Clinical characteristics and causative pathogens in varied FUBC-timing groups

A total of 1,247 adults were categorized as five groups based on the FUBC timing related to AAT: 65 (5.2%) with FUBCs prior to AAT, 202 (16.2%) at 0–3 days, 470 (37.7%) at 3.1–6 days, 299 (24.0%) at 6.1–9 days, and 211 (16.9%) at >9 days of AAT (Fig. 1). Clinical characteristics and outcomes among five groups were compared in Table 2. The similarity of patient demography (the elderly, gender, and nursing-home residents), comorbidities, comorbidity severity, and bacteremia severity at onset, was exhibited. However, there were significant differences in three bacteremia sources (primary bacteremia, vascular catheter-related infections, and infective endocarditis), the timing of FUBC sampling (*i.e*., time-to-FUBC) and AAT administration (*i.e*., time-to-AAT), the duration of hospital and ICU stay, and 15-day and 30-day crude mortality rate among five groups. Of a total of 1,409 aerobic isolates, the distribution of causative pathogens was shown in Table 3, and the significant variation was present in streptococci and *Acinetobacter* species among five groups.

**Table 2:** Clinical characteristics and patient outcomes in different follow-up blood culture (FUBC) subgroups.

Variables	Patients numbers (%)					*P* values
	Prior to AAT n = 65	0–3 days of AAT n = 202	3.1–6 days of AAT n = 470	6.1–9 days of AAT n = 299	>9 days of AAT n = 211	
Gender, male	35 (53.8)	109 (54.0)	263 (56.0)	167 (55.9)	124 (58.8)	0.89
The elderly, ≥65 years	34 (52.3)	112 (55.4)	286 (60.9)	170 (56.9)	131 (62.1)	0.37
Nursing-home residents	5 (7.7)	16 (7.9)	35 (7.4)	19 (6.4)	12 (5.7)	0.88
Fatal comorbidities (McCabe classification)	23 (35.4)	56 (27.7)	135 (28.7)	101 (33.8)	65 (30.8)	0.44
Comorbidities						
Hypertension	31 (47.7)	104 (51.5)	249 (53.0)	146 (48.8)	95 (45.0)	0.38
Malignancies	25 (38.5)	69 (34.2)	142 (30.2)	98 (32.8)	78 (37.0)	0.39
Diabetes mellitus	24 (36.9)	79 (39.1)	172 (36.6)	114 (38.1)	97 (46.0)	0.23
Neurological diseases	20 (30.8)	51 (25.2)	134 (28.5)	77 (25.8)	55 (26.1)	0.80
ESRD on regular hemodialysis	6 (9.2)	26 (12.9)	48 (10.2)	26 (8.7)	24 (11.4)	0.63
Liver cirrhosis	4 (6.2)	18 (8.9)	54 (11.5)	45 (15.1)	30 (14.2)	0.11
Polymicrobial episodes	11 (16.9)	22 (10.9)	45 (9.6)	34 (11.4)	16 (7.6)	0.24
Bacteremia severity at onset						
Pitt bacteremia score ≥4	10 (15.4)	41 (20.3)	98 (20.9)	63 (21.1)	60 (28.4)	0.11
ICU admission through EDs	11 (16.9)	31 (15.3)	63 (13.4)	53 (17.7)	44 (20.9)	0.15
Major bacteremia sources						
Urinary tract infections	18 (27.7)	38 (18.8)	92 (19.6)	65 (21.7)	47 (22.3)	0.52
Primary bacteremia	11 (16.9)	14 (6.9)	48 (10.2)	23 (7.7)	6 (2.8)	0.001
Pneumonia	9 (13.8)	31 (15.3)	91 (19.4)	63 (21.1)	46 (21.8)	0.32
Vascular catheter-related infections	9 (13.8)	22 (10.9)	31 (6.6)	10 (3.3)	5 (2.4)	<0.001
Soft-tissue infections	7 (10.8)	24 (11.9)	67 (14.3)	50 (16.7)	38 (18.0)	0.30
Intra-abdominal infections	3 (4.6)	25 (12.4)	44 (9.4)	24 (8.0)	22 (10.4)	0.32
Infective endocarditis	2 (3.1)	24 (11.9)	38 (8.1)	13 (4.3)	13 (6.2)	0.01
Bone and joint infections	1 (1.5)	16 (7.8)	43 (9.1)	25 (8.4)	18 (8.5)	0.35
Length, mean ± standard deviation						
Time-to-FUBC, day	4.6 ± 2.2	3.6 ± 2.2	4.5 ± 1.8	7.6 ± 1.7	15.6 ± 6.3	<0.001
Time-to-AAT, hour	179.2 ± 140.0	37.1 ± 41.6	10.6 ± 24.4	12.1 ± 25.4	11.0 ± 24.8	<0.001
Time-to-defervescence, day	9.9 ± 12.0	9.8 ± 9.7	11.7 ± 25.0	12.1 ± 9.6	11.8 ± 9.7	0.69
Total hospitalization, day	18.8 ± 16.1	22.1 ± 19.9	23.7 ± 23.3	26.0 ± 20.4	39.8 ± 34.7	<0.001
ICU stay, day	2.7 ± 11.9	3.5 ± 11.9	3.3 ± 9.8	3.8 ± 9.7	7.3 ± 15.7	<0.001
Crude mortality rate						
15-day	6 (9.2)	21 (10.4)	26 (5.5)	17 (5.7)	8 (3.8)	0.047
30-day	10 (15.4)	37 (18.3)	46 (9.8)	39 (13.0)	25 (11.8)	0.04

AAT = appropriate antibiotic therapy; ICU = Intensive care unit; ESRD = end-stage renal disease.

**Table 3:** Causative pathogens of community-onset bacteremia in different follow-up blood culture (FUBC) subgroups.

Causative pathogens	Isolate number (% of total isolates in the FUBC subgroup)					*P* values
	Prior to AAT n = 78	0–3 days of AAT n = 227	3.1–6 day of AAT n = 525	6.1–9 day of AAT n = 345	≥9 day of AAT n = 234	
*Staphylococcus aureus*	19 (24.4)	58 (25.6)	113 (21.5)	67 (19.4)	45 (19.2)	0.38
*Escherichia coli*	17 (21.8)	37 (16.3)	123 (23.4)	87 (25.2)	60 (25.6)	0.10
*Klebsiella* species	6 (7.7)	28 (12.3)	78 (14.9)	46 (13.3)	41 (17.5)	0.21
*Streptococcus* species	5 (6.4)	45 (19.8)	112 (21.3)	51 (14.8)	24 (10.3)	<0.001
*Pseudomonas* species	5 (6.4)	8 (3.5)	18 (3.4)	9 (2.6)	11 (4.7)	0.46
*Acinetobacter* species	5 (6.4)	4 (1.8)	8 (1.5)	3 (0.9)	2 (0.9)	0.008
*Enterococcus* species	4 (5.1)	10 (4.4)	18 (3.4)	16 (4.6)	9 (3.8)	0.88
*Enterobacter* species	3 (3.8)	8 (3.5)	11 (2.1)	7 (2.0)	4 (1.7)	0.59
*Salmonella* species	0 (0)	4 (1.8)	11 (2.1)	8 (2.3)	7 (3.0)	0.60
*Proteus* species	0 (0)	1 (0.4)	7 (1.3)	7 (2.0)	5 (2.1)	0.35

### Impacts of bacterial growth in FUBCs among varied FUBC-timing groups

Of 65 patients with FUBCs prior to AAT, clinical predictors of 30-day mortality through the univariate analyses included bacteremic pneumonia and nursing-home residents (Table 4). In the multivariate analysis, two independent factors of 30-day mortality, fatal comorbidities (McCabe classification) and nursing-home residents, were recognized. Of note, the impact of bacterial growth in FUBCs on mortality was trivial (AOR, 2.86; *P* = 0.25).

**Table 4:** Risk factors of 30-day crude mortality in patients with follow-up blood culture (FUBC) sampled at different time related to appropriate antibiotic therapy (AAT).

Variables	Patient number (%)		Univariate analysis		Multivariate analysis	
	Death	Survival	OR (95% CI)	*P* value	Adjusted OR (95% CI)	*P* value
**FUBCs prior to AAT (n** = **65)**	**n** = **10**	**n** = **55**				
Fatal comorbidities (McCabe classification)	6 (60.0)	17 (30.9)	3.35 (0.84 = 13.44)	0.15	6.81 (1.02–45.41)	0.048
Pitt bacteremia score ≥4 at onset	3 (30.0)	7 (12.7)	2.94 (0.61–14.10)	0.18	1.00 (0.13–7.46)	1.00
Bacteremic pneumonia	4 (40.0)	5 (9.1)	6.67 (1.40–31.85)	0.03	6.33 (0.77–51.90)	0.09
Nursing-home residents	3 (30.0)	2 (3.6)	11.36 (1.61–80.24)	0.02	12.80 (1.24–132.27)	0.03
Bacterial growth in FUBCs	5 (50.0)	15 (27.3)	2.67 (0.68–10.54)	0.26	2.86 (0.49–16.84)	0.25
**FUBCs sampled at 0–3 day’s AAT (n** = **202)**	**n** = **37**	**n** = **165**				
Fatal comorbidities (McCabe classification)	22 (59.5)	34 (20.6)	5.65 (2.65–12.04)	<0.001	3.79 (1.23–11.66)	0.02
Comorbid malignancies	20 (54.1)	49 (29.7)	2.79 (1.35–5.77)	0.005	1.48 (0.48–4.59)	0.50
Pitt bacteremia score ≥4 at onset	19 (51.4)	22 (13.3)	6.86 (3.13–15.05)	<0.001	5.13 (1.93–13.65)	0.001
*Pseudomonas* bacteremia	5 (13.5)	3 (1.8)	8.44 (1.92–37.09)	0.006	2.26 (0.33–15.65)	0.41
Bacteremia sources						
Pneumonia	14 (37.8)	17 (10.3)	5.30 (2.31–12.19)	<0.001	2.83 (0.93–8.57)	0.07
Urinary tract infections	1 (2.7)	37 (22.4)	0.10 (0.01–0.73)	0.006	0.12 (0.01–1.05)	0.06
Inadequate source control	4 (10.8)	5 (3.0)	3.88 (0.99–15.22)	0.06	3.36 (0.64–17.63)	0.15
Bacterial growth in FUBCs	7 (18.9)	62 (37.6)	0.39 (0.16–0.94)	0.03	0.39 (0.14–1.11)	0.08
**FUBCs sampled at 3.1–6 day’s AAT (n** = **470)**	**n** = **46**	**n** = **424**				
Inappropriate empirical antimicrobial therapy	11 (23.9)	41 (9.7)	2.94 (1.39–6.22)	0.003	2.29 (0.97–5.40)	0.06
Nursing-home residents	8 (17.4)	27 (6.4)	3.10 (1.32–7.29)	0.01	1.34 (0.45–3.96)	0.60
Fatal comorbidities (McCabe classification)	21 (45.7)	114 (26.9)	2.28 (1.23–4.24)	0.008	1.56 (0.71–3.43)	0.27
Comorbidities						
Liver cirrhosis	9 (19.6)	45 (10.6)	2.05 (0.93–4.52)	0.07	2.22 (0.91–5.41)	0.08
Malignancies	22 (47.8)	120 (28.3)	2.32 (1.25–4.30)	0.006	2.33 (1.05–5.15)	0.04
Neurological diseases	20 (43.5)	114 (26.9)	2.09 (1.12–3.89)	0.02	1.99 (0.92–4.28)	0.08
Pitt bacteremia score ≥4 at onset	19 (41.3)	79 (18.6)	3.07 (1.63–5.80)	<0.001	3.20 (1.56–6.54)	0.001
Bacterial growth in FUBCs	16 (34.8)	55 (13.0)	3.58 (1.83–6.99)	<0.001	3.75 (1.80–7.79)	<0.001
**FUBCs sampled at 6.1–9 day’s AAT (n = 299)**	**n = 39**	**n = 260**				
Fatal comorbidities (McCabe classification)	20 (51.3)	81 (31.2)	2.33 (1.18–4.59)	0.01	0.55 (0.19–1.56)	0.26
Comorbidities						
Hypertension	13 (33.3)	133 (51.2)	0.48 (0.24–0.97)	0.04	0.84 (0.36–1.96)	0.68
Diabetes mellitus	7 (17.9)	107 (41.2)	0.32 (0.13–0.74)	0.005	0.29 (0.10–1.02)	0.06
Liver cirrhosis	11 (28.2)	34 (13.1)	2.61 (1.19–5.73)	0.01	4.44 (1.57–12.60)	0.005
End-stage renal diseases	0 (0)	26 (10.0)	—	0.03	—	1.00
Malignancies	25 (64.1)	73 (28.1)	4.57 (2.25–9.29)	<0.001	3.83 (1.40–10.49)	0.009
Pitt bacteremia score ≥4 at onset	16 (41.0)	47 (18.1)	3.15 (1.55–6.43)	0.001	3.08 (1.22–7.78)	0.02
Polymicrobial bacteremia	8 (20.5)	26 (10.0)	2.32 (0.97–5.58)	0.06	1.64 (0.54–5.01)	0.38
Causative microorganisms						
*Pseudomonas* species	4 (10.3)	5 (1.9)	5.83 (1.49–22.74)	0.02	2.14 (0.25–18.61)	0.49
*Enterobacter* species	3 (7.7)	4 (1.5)	5.33 (1.15–24.81)	0.05	28.07 (2.48–317.46)	0.007
*Klebsiella* species	10 (25.6)	36 (13.8)	2.15 (0.96–4.78)	0.06	1.45 (0.54–3.89)	0.46
Bacteremia sources						
Pneumonia	17 (43.6)	46 (17.7)	3.60 (1.777–7.30)	<0.001	3.84 (1.51–9.77)	0.005
Bone and joint infections	0 (0)	25 (9.6)	–	0.06	–	1.00
Bacterial growth in FUBCs	4 (10.3)	12 (4.6)	2.36 (0.72–7.73)	0.14	2.19 (0.46–10.36)	0.32
**FUBCs sampled at >9 day’s AAT (n** = **211)**	**n** = **25**	**n** = **186**				
Comorbidities						
Diabetes mellitus	7 (28.0)	90 (48.4)	0.42 (0.17–1.04)	0.06	0.48 (0.18–1.28)	0.14
Malignancies	13 (52.0)	65 (34.9)	2.02 (0.87–4.67)	0.097	1.72 (0.70–4.22)	0.24
Neurological diseases	3 (12.0)	52 (28.0)	0.35 (0.10–1.22)	0.096	0.25 (0.07–1.00)	0.05
Pitt bacteremia score ≥4 at onset	12 (48.0)	48 (25.8)	2.65 (1.13–6.21)	0.04	2.78 (1.08–7.17)	0.04
Bacteremic pneumonia	9 (36.0)	37 (19.9)	2.27 (0.93–5.53)	0.07	1.97 (0.73–5.31)	0.18
Bacterial growth in FUBCs	1 (4.0)	22 (11.8)	0.31 (0.04–2.41)	0.24	0.41 (0.05–3.43)	0.41

CI = confidence interval; OR = odds ratio.

Among 202 patients with FUBCs sampled at 0–3 days of AAT, the positive predictors of 30-day mortality recognized by the univariate analysis included fatal comorbidities, comorbid malignancies, a critical illness (Pitt bacteremia score ≥ 4) at bacteremia onset, *Pseudomonas* bacteremia, and bacteremic pneumonia, and a negative predictor was bacteremia due to urinary tract infections (Table 4). Although bacterial growth in FUBCs was linked to 30-day mortality (OR, 0.39, *P* = 0.03) in the univariate analysis, the association of bacterial growth of FUBCs and 30-day mortality was not evident (AOR, 0.39, *P* = 0.08), after adjusting independent predictors in the multivariable regression model.

For 470 patients with FUBCs sampled at 3.1–6 days of AAT (Table 4), the predictors of 30-day mortality recognized by the univariate analysis included inappropriate empirical antimicrobial therapy, nursing-home residents, fatal comorbidities, comorbidities of malignancies or neurological diseases, a critical illness at bacteremia onset, and bacterial growth of FUBCs. Most importantly, the adverse impact of bacterial growth of FUBCs remained significant (AOR, 3.75, *P* < 0.001) in the multivariate regression model.

Among 299 patients with FUBCs sampled at 6.1–9 days of AAT, the significant predictors of 30-day mortality in the univariate test included fatal comorbidities, comorbidities of hypertension, diabetes mellitus, liver cirrhosis, end-stage renal diseases, or malignancies, a critical illness at bacteremia onset, *Pseudomonas*, *Enterobacter*, or *Klebsiella* bacteremia, and bacteremic pneumonia (Table 4). Of note, the impact of bacterial growth of FUBCs on 30-day mortality was not significant in the univariate analysis (OR, 2.36, *P* = 0.14) and multivariate regression model (AOR, 2.19, *P* = 0.32).

For 211 patients with FUBCs sampled after 9 days of AAT, the only predictor of 30-day mortality by the univariate analysis was a critical illness at bacteremia onset (Table 4); and bacterial growth of FUBCs was not associated with 30-day mortality in the univariate analysis (OR, 0.31, *P* = 0.24) and multivariate regression model (AOR, 0.41, *P* = 0.41).

## Discussion

Generally, clinicians agree that the best way to achieve a rapid onset of antibacterial action in antimicrobial therapy is through intravenous administration of antibiotics. However, over the past 20 years, economic pressure had changed the medical culture from conventional intravenous therapy for the entire therapeutic course to early oral switch or home parenteral administration of antibiotics. Therefore, many clinical trials have comprehensively studied the role of FUBCs in specific pathogens and bacteremia foci. For example, FUBCs have been recommended for *S. aureus* bacteremia^2,3^ and infective endocarditis^3,18^ but appeared to be unnecessary in the management of *Klebsiella pneumoniae*^19^ or Gram-negative bacillary bacteremia^4,5^. In addition, the evidence supporting that bacterial growth in FUBCs among patients receiving active antimicrobial therapy results in a poor prognosis was lacking, and little is known for the optimal timing of FUBC sampling after the initiation and AAT. Herein, focusing on community-onset bacteremia, the differential impact of bacterial growth in FUBCs on patient outcomes by variable timing of FUBCs was addressed.

Generally speaking, breakthrough bacteremia was regarded as the development of bacteremic episode despite therapeutic administration of antimicrobials active *in vitro* against the causative organism^20^. Such a clinical setting is linked to an increasing risk of death and a need of prolonged antibiotic therapy and hospitalization^20,21^. However, there is no current consensus regarding the duration of treatment or timing of FUBCs to detect breakthrough bacteremia. Herein, we focused on the time lapse between antimicrobial administration and steady serum drug concentrations for optimization of antimicrobial therapy, and thus the cutoff point selected was 3 days after AAT. More importantly, the episodes of breakthrough bacteremia by FUBCs found in 3.1 to 6 days after AAT had the most significant prognostic impact in our cohort, suggestive of the cost-effective timing of FUBCs in the cases of community-onset bacteremia.

In the literature, FUBC sampling has been recommended as a standard of care to reduce the case fatality rate of *S. aureus* bacteremia^2,3^, but not for Gram-negative bacillary bacteremia^4,5^. However, bacterial growth in FUBCs was most commonly found in the cases of *Enterobacter* bacteremia (36.4%) herein. Such a discrepancy might come from the fact that essential determinants of FUBC growth, such as bacteremia sources, adequacy of source control, timing of ATT, and bacteremia severity, were not compressively concerned in published reports, but were analyzed in the present study.

Several limitations are inherent in the study design. First, medical records could not exploit the reasons why FUBCs were ordered in approximately one third of hospitalized adults with community-onset bacteremia. Because the distribution of causative pathogens in those with FUBCs was similar to that in our published cohort^10^, the concern for selection bias might be neglected. Second, the dissimilarity of bacteremia sources, causative pathogens, and the duration of hospitalization was observed in various timing groups of FUBC sampling, but in the multivariate regression model, these confounding factors were controlled to reduce their interference with the prognostic effects of FUBC growth. Finally, the practices and indications for FUBC samplings might vary among hospitals and the indications of FUBC sampling in our study were not predetermined. We did not investigate the sampling frequency of FUBCs among varied bacteremia sources and causative pathogens, but showed the data of persistent bacteremia in specific subgroups with indicated FUBCs. Therefore, our results cannot be extrapolated to directly reflect the needs of FUBCs for specific infection sources (such as bone and joint infections) or causative pathogens (such as *Enterobacter* or *Acinetobacter* bacteremia). More prospective clinical studies with indicated FUBCs are warranted to reveal clinical utility of routine FUBCs.

Conclusively, the present study indicates that appropriate timing of FUBCs can find the prognostic significance of bacterial growth in FUBCs in the management of community-onset bacteremia. Accordingly, FUBC sampling at 3–6 days after the initiation of AAT is suggested.

## Data Availability

All data was available on requirement.

## References

[1] Bates DW, Pruess KE, Lee TH (1995). How bad are bacteremia and sepsis? Outcomes in a cohort with suspected bacteremia. Archives of internal medicine.

[2] Chong YP (2013). Treatment duration for uncomplicated Staphylococcus aureus bacteremia to prevent relapse: analysis of a prospective observational cohort study. Antimicrob Agents Chemother.

[3] Wiggers JB, Xiong W, Daneman N (2016). Sending repeat cultures: is there a role in the management of bacteremic episodes? (SCRIBE study). BMC Infect Dis.

[4] Canzoneri CN, Akhavan BJ, Tosur Z, Andrade PEA, Aisenberg GM (2017). Follow-up Blood Cultures in Gram-Negative Bacteremia: Are They Needed?. Clin Infect Dis.

[5] Harris JA, Cobbs CG (1973). Persistent gram-negative bacteremia. Observations in twenty patients. Am J Surg.

[6] Washington JA, Ilstrup DM (1986). Blood cultures: issues and controversies. Rev Infect Dis.

[7] Aronson MD, Bor DH (1987). Blood cultures. Ann Intern Med.

[8] von Elm E (2007). The Strengthening the Reporting of Observational Studies in Epidemiology (STROBE) statement: guidelines for reporting observational studies. Lancet.

[9] Lee, C. C. *et al*. Clinical benefit of appropriate empirical fluoroquinolone therapy for adults with community-onset bacteremia in comparison with third-generation-cephalosporin therapy. *Antimicrob Agents Chemother* **61**, 10.1128/AAC.02174-16 (2017). 10.1128/AAC.02174-16 PMC5278695 27855072

[10] Lee CC, Lee CH, Hong MY, Tang HJ, Ko WC (2017). Timing of appropriate empirical antimicrobial administration and outcome of adults with community-onset bacteremia. Crit Care.

[11] Lee CC (2007). Clinical significance of potential contaminants in blood cultures among patients in a medical center. J Microbiol Immunol Infect.

[12] Gilbert, D. N., Moellering, R. C. Jr., Eliopoulos, G. M., Chambers, H. F., & Saag, M. S. Selected pharmacologic faetures of antimicrobial agents. *The Sanford Guide to Antimicrobial Therapy*, 78–82 (2009).

[13] Clinical and Laboratory Standards Institute. Performance standards for antimicrobial susceptibility testing; approved standard. Twenty-sixth informational supplement. CLSI Document M100-S26. Wayne, PA: CLSI, 2016 (2016).

[14] Leibovici L (1998). The benefit of appropriate empirical antibiotic treatment in patients with bloodstream infection. J Intern Med.

[15] Chotiprasitsakul D (2018). Comparing the outcomes of adults with Enterobacteriaceae bacteremia receiving short-course versus prolonged-course antibiotic therapy in a multicenter, propensity score-matched cohort. Clin Infect Dis.

[16] Schellevis, F. G., van der Velden, J., van de Lisdonk, E., van Eijk, J. T. & van Weel, C. Comorbidity of chronic diseases in general practice. *J Clin Epidemiol* **46**, 469-473, 0895-4356(93)90024-U [pii] (1993). 10.1016/0895-4356(93)90024-u 8501473

[17] McCabe WR (1974). Gram-negative bacteremia. Adv Intern Med.

[18] Chandrasekar PH, Brown WJ (1994). Clinical issues of blood cultures. Arch Intern Med.

[19] Kang CK (2013). Can a routine follow-up blood culture be justified in Klebsiella pneumoniae bacteremia? A retrospective case-control study. BMC Infect Dis.

[20] Weinstein MP, Reller LB (1984). Clinical importance of “breakthrough” bacteremia. Am J Med.

[21] Tsai MH (2015). Breakthrough bacteremia in the neonatal intensive care unit: incidence, risk factors, and attributable mortality. Am J Infect Control.

